# Number of Candidate Effector Genes in Accessory Genomes Differentiates Pathogenic From Endophytic *Fusarium oxysporum* Strains

**DOI:** 10.3389/fpls.2021.761740

**Published:** 2021-11-29

**Authors:** Maria E. Constantin, Like Fokkens, Mara de Sain, Frank L. W. Takken, Martijn Rep

**Affiliations:** Molecular Plant Pathology, Faculty of Science, Swammerdam Institute for Life Sciences, University of Amsterdam, Amsterdam, Netherlands

**Keywords:** effectors, endophyte, *Fusarium oxysporum*, *miniature transposable element*, pathogen, comparative genomics

## Abstract

The fungus *Fusarium oxysporum* (Fo) is widely known for causing wilt disease in over 100 different plant species. Endophytic interactions of Fo with plants are much more common, and strains pathogenic on one plant species can even be beneficial endophytes on another species. However, endophytic and beneficial interactions have been much less investigated at the molecular level, and the genetic basis that underlies endophytic versus pathogenic behavior is unknown. To investigate this, 44 Fo strains from non-cultivated Australian soils, grass roots from Spain, and tomato stems from United States were characterized genotypically by whole genome sequencing, and phenotypically by examining their ability to symptomlessly colonize tomato plants and to confer resistance against Fusarium Wilt. Comparison of the genomes of the validated endophytic Fo strains with those of 102 pathogenic strains revealed that both groups have similar genomes sizes, with similar amount of accessory DNA. However, although endophytic strains can harbor homologs of known effector genes, they have typically fewer effector gene candidates and associated non-autonomous transposons (*mimps*) than pathogenic strains. A pathogenic ‘lifestyle’ is associated with extended effector gene catalogs and a set of “host specific” effectors. No candidate effector genes unique to endophytic strains isolated from the same plant species were found, implying little or no host-specific adaptation. As plant-beneficial interactions were observed to be common for the tested Fo isolates, the propensity for endophytism and the ability to confer biocontrol appears to be a predominant feature of this organism. These findings allow prediction of the lifestyle of a Fo strain based on its genome sequence as a potential pathogen or as a harmless or even beneficial endophyte by determining its effectorome and *mimp* number.

## Introduction

Until the 19th century the interior of plants was generally considered to be practically sterile. Plant–microbe interactions were thought to be limited to pathogenic microorganisms causing visible disease symptoms on the plant (Fernbach, 1888; Hardoim et al., 2015). Nowadays, a large body of research in plant microbiomes provides a glimpse of the scale and complexity of interactions between plants and microorganisms. Microorganisms inhabiting plant tissues can be broadly divided into pathogens and endophytes, based on their capability to cause disease, or not, to their host. Predicting the potential influence of plant-associated microorganisms on plant performance is an important next step, especially since some endophytes can provide benefits to the colonized host plant in terms of growth and/or disease protection (Rodriguez et al., 2009; Busby et al., 2016), which makes their use in agriculture highly attractive.

Whether a plant–microbe interaction culminates in disease depends – among other factors – on the host. Some fungi initially characterized as pathogens on one plant species have been found to colonize other plant species asymptomatically as endophytes (Gordon et al., 1989; Redman et al., 2001; Malcolm et al., 2013; van Kan et al., 2014; van der Does et al., 2018). The biotic environment also plays a role: when combined with a (consortium of) other microorganism(s) a pathogen can fail to cause disease (Duran et al., 2018). Finally, microbes may differ in their propensity to cause disease independent of host susceptibility and environmental factors.

*Fusarium oxysporum* is an example of a pathogen that is also a common asymptomatic inhabitant of plant roots (Gordon et al., 1989; Inami et al., 2014; Pereira et al., 2019). Members of this species complex are well-known as devastating pathogens causing wilt disease in more than 100 plant species, and pathogenic strains are grouped into ff. spp. based on their host range (Edel-Hermann and Lecomte, 2019). Notably, Fo pathogens can colonize non-susceptible hosts asymptomatically as endophytes (Smith and Snyder, 1975; Pereira et al., 2019). The widespread habitat of Fo, its dual lifestyle, and the fact that many whole genome sequences are available for this species, makes Fo a suitable model to study the genetic basis of pathogenic and endophytic potential.

*Fusarium oxysporum* genomes are compartmentalized into core chromosomes that are highly similar among Fo strains, and accessory chromosomes that are not. Accessory chromosomes are enriched in transposable elements and dispensable for viability of the fungus. Some accessory chromosomes are associated with pathogenicity and are referred to as pathogenicity chromosomes (Ma et al., 2010; Vlaardingerbroek et al., 2016; van Dam et al., 2017; Armitage et al., 2018; Fokkens et al., 2018). Transfer of one or more pathogenicity chromosomes from a pathogenic strain to the endophytic strain Fo47 confers pathogenicity to the latter, with the same host range as the original pathogen (Ma et al., 2010; van Dam et al., 2017; Li et al., 2020).

In the case of tomato pathogen Fo f. sp. *lycopersici* (Fol) strain 4287, its pathogenicity chromosome (chromosome 14) is particularly enriched for class II (DNA) transposable elements, some of which are associated with genes involved in pathogenicity, such as effector genes. Effector genes encode small *in planta* secreted proteins that are proposed to manipulate the host to promote colonization. Specifically, the non-autonomous DNA transposon *miniature impala* (*mimp*) is associated with promoters of effector genes in Fol (Schmidt et al., 2013). Fourteen effector proteins have been identified in the xylem sap of tomato plants infected with Fol and some of these have been shown to contribute to pathogenicity toward tomato (Rep et al., 2004; Houterman et al., 2007, 2009; Schmidt et al., 2013; Ma et al., 2015). The consistent presence of *mimps* in the promoter region of these *SIX* genes and some other virulence-associated genes provides the means to predict candidate effector genes in different Fo genomes (Schmidt et al., 2016; van Dam et al., 2016). Fo strains that are pathogenic toward the same host have a similar set of effectors, presumably enabling them to cause disease symptoms on that host (van Dam et al., 2016).

It is unclear whether endophytic Fo strains have a similar specialization in terms of effectors and genomic organization. Previous research on two non-pathogenic Fo strains (Fo47 and FoMN14) suggests that their compartmentalized genomes contain fewer *mimps* and candidate effector genes (van Dam et al., 2016, 2017). Due to the limited number of endophyte genomes analyzed, however, this conclusion remains tentative. Whether endophytes contain specialized ‘endophyte-effectors’ or whether there is a qualitative or quantitative genetic difference at all between ‘endophytes’ and ‘pathogens’ remained to be investigated.

To determine genomic signatures of Fo endophytes, we selected 44 Fo strains isolated from soil or from asymptomatic plants from diverse habitats on three continents and sequenced their genomes. In addition, we tested the pathogenicity and ability of these strains to colonize tomato and confer resistance to disease caused by a pathogenic Fol strain. By comparing this dataset with >100 publicly available Fo genomes, we observe that the genomes of Fo endophytes are similar to genomes of Fo pathogens in terms of size of core and accessory material. However, Fo endophytes contain fewer *mimps* and fewer candidate effector genes located on their accessory genome than pathogenic Fo strains. This suggests that the number of *mimps* and candidate effector genes can be used to predict whether a Fo strain has the potential to cause disease or is a probable endophyte.

## Materials and Methods

### Pathogenicity Testing of *Fusarium oxysporum* Strains and Disease Assays

The tomato (*Solanum lycopersicum*) variety C32, susceptible to all three known Fol races, was used for disease assays with the selected Fo strains. Tomato plants were grown in a climate-controlled greenhouse with a day–night temperature of 25°C, 16 h light/8 h dark and a relative humidity of 65%. Fo strains were selected based on their geographical location (Table 1) and grown on Potato Dextrose Agar (PDA) plates at 25°C for 7–10 days in the dark. To obtain spores, one piece of agar from PDA plates was transferred to 100 ml of minimal media (1% KNO_3_, 3% sucrose, 0.17% Yeast Nitrogen Base without amino acids and ammonia) and incubated for 3–5 days at 25°C, 150 rpm. To obtain spores of Fo5, Fo16, and Fo29 strains 1% mung bean media (Garcia-Bastidas et al., 2019) was used instead of the minimal media, incubated 3–5 days at 25°C, 150 rpm. Fo18, Fo25, Fo28, and Fo39 from the Spanish isolate collection did not produce sufficient spores in minimal media and/or mung bean media and therefore their ability for tomato colonization and pathogenicity could not be tested.

**TABLE 1:** Endophytic and environmental strains of *F. oxysporum* investigated in this study.

sFP	Strain	Original designation	Isolated from	Tomato stem outgrowth	Origin of strain	References
3804	Fo1	RBG1687	*Wollemia nobilis* leaves	(3/5)	Australia	Rocha et al., 2016
3805	Fo2	RBG1693	*Actinotus helianthi* roots	(5/5)	Australia	Rocha et al., 2016
3806	Fo3^+^	RBG5713	Soil	(1/5)	Australia	Rocha et al., 2016
3807	Fo4	RBG5786	Soil	(1/5)	Australia	Rocha et al., 2016
3808	Fo5	RBG5789	Soil	(1/5)	Australia	Rocha et al., 2016
3809	Fo6	RBG5791	Soil	(0/5)	Australia	Rocha et al., 2016
3810	Fo7	RBG5798	Soil	(0/5)	Australia	Rocha et al., 2016
3811	Fo8	RBG5820	Soil	(3/5)	Australia	Rocha et al., 2016
3812	Fo9	RBG5824	Soil	(5/5)	Australia	Rocha et al., 2016
3813	Fo10^+^	RBG5827	Soil	(3/5)	Australia	Rocha et al., 2016
3814	Fo11	RBG5831	Soil	(4/5)	Australia	Rocha et al., 2016
3815	Fo12	RBG5835	Soil	(5/5)	Australia	Rocha et al., 2016
3816	Fo13^+^	RBG5843	Soil	(1/5)	Australia	Rocha et al., 2016
3817	Fo14^+^	RBG5856	Soil	(3/5)	Australia	Rocha et al., 2016
3818	Fo15	RBG5870	Soil	(3/5)	Australia	Rocha et al., 2016
3895	Fo16	Th7a	*Festuca rubra* roots	(0/5)	Spain	Pereira et al., 2019
3896	Fo17	Th13a	*Festuca rubra* roots	(4/5)	Spain	Pereira et al., 2019
3897	Fo18	Th21a	*Festuca rubra* roots	n.d.	Spain	Pereira et al., 2019
3899	Fo20	Th20a	*Festuca rubra* roots	(3/5)	Spain	Pereira et al., 2019
3910	Fo24	Th1b	*Festuca rubra* roots	(1/5)	Spain	Pereira et al., 2019
3919	Fo25	Th18a	*Festuca rubra* roots	n.d.	Spain	Pereira et al., 2019
3920	Fo26	Cd5a	*Festuca rubra* roots	(1/5)	Spain	Pereira et al., 2019
3902	Fo28	Cd1b	*Festuca rubra* roots	n.d.	Spain	Pereira et al., 2019
3911	Fo29	Cd10b	*Festuca rubra* roots	(0/5)	Spain	Pereira et al., 2019
3906	Fo35	Cd13b	*Festuca rubra* roots	(3/5)	Spain	Pereira et al., 2019
3909	Fo39	Eb7b	*Festuca rubra* roots	n.d.	Spain	Pereira et al., 2019
3917	Fo41	Eb1b	*Festuca rubra* roots	(4/5)	Spain	Pereira et al., 2019
3916	Fo44	Eb18a	*Festuca rubra* roots	(3/5)	Spain	Pereira et al., 2019
3915	Fo45	Eb11b	*Festuca rubra* roots	(5/5)	Spain	Pereira et al., 2019
3923	Fo46	FL1st4h22	*Solanum lycopersicum* stem	(0/5)	United States	Demers et al., 2015
3922	Fo48^+^	2-2st2h8	*Solanum lycopersicum* stem	(4/5)	United States	Demers et al., 2015
3924	Fo49^+^	Hz4c1h8	*Solanum lycopersicum* crown	(2/3)	United States	Demers et al., 2015
3925	Fo52	MF4st2h5	*Solanum lycopersicum* stem	(3/5)	United States	Demers et al., 2015
3926	Fo53	FL1c1h5	*Solanum lycopersicum* crown	(1/5)	United States	Demers et al., 2015
3927	Fo54*^+^	3-4st4h14	*Solanum lycopersicum* stem	(5/5)	United States	Demers et al., 2015
3914	Fo57	2-4st1h13	*Solanum lycopersicum* stem	(2/5)	United States	Demers et al., 2015
3929	Fo58	2-1c1h13	*Solanum lycopersicum* crown	(1/5)	United States	Demers et al., 2015
3930	Fo59*	1-2c1h12	*Solanum lycopersicum* crown	(3/5)	United States	Demers et al., 2015
3931	Fo63	FL3st2h4	*Solanum lycopersicum* stem	(0/5)	United States	Demers et al., 2015
3921	Fo65	3-4st1h2	*Solanum lycopersicum* stem	(2/5)	United States	Demers et al., 2015
3932	Fo68	2-3c3h21	*Solanum lycopersicum* crown	(0/5)	United States	Demers et al., 2015
3933	Fo69	3-2c2h20	*Solanum lycopersicum* crown	(0/5)	United States	Demers et al., 2015
3934	Fo74	GM4St4h5	*Solanum lycopersicum* stem	(0/5)	United States	Demers et al., 2015
3935	Fo75	1-4st2h13	*Solanum lycopersicum* stem	(0/5)	United States	Demers et al., 2015
730	Fo47	Fo47	Soil	(4/5)	France	Lemanceau and Alabouvette, 1991

* Strains that can occasionally induce mild disease symptoms in tomato. ^+^ Strains with EF1alfa sequence different than previously described.

*Fusarium oxysporum* strains were tested for pathogenicity and their ability to confer biocontrol toward the wild-type pathogen Fol4287 (sFP801) (Di Pietro et al., 2003). This was done by inoculating 10-day-old tomato seedlings, with trimmed roots, for 5 min in either a suspension of 10^7^ Fo spores/ml or 10^7^ spores/ml: 10^7^ spores/ml (ratio 1:1 Fo/Fol4287) for co-inoculation treatments. Subsequently, tomato seedlings were potted and disease index and fresh weight was assessed 3 weeks after inoculation. Disease was scored as described (Constantin et al., 2019), in short, vascular browning at the cotyledon level was assessed, where 0 = no symptoms; 1 = brown vessel(s) at the crown, but not at the cotyledon level; 2 = one or two brown vessels at the cotyledon level; 3 = at least three brown vessels and growth distortion of the plant, 4 = all vessels brown or the plant is small and wilted, 5 = dead plant. Strains Fo1, Fo3, Fo4, Fo8, Fo10, Fo11, Fo17, and Fo57 were tested again in a second inoculation assay, in which the observation that Fo3 protects tomato plants less well than Fo47 was confirmed (Supplementary Figure 1).

### Fungal Recovery Assay

To assess the colonization potential of Fo in tomato, 3 weeks after root inoculation five tomato stems per treatment were collected and surfaced sterilized for approximately 4 min in 70% ethanol. The ethanol was removed and stems were rinsed twice with sterile water. The extremities of each stem were removed, and the central piece of a cotyledon and crown part of the stem was placed on PDA plates containing 200 mg/l streptomycin and 100 mg/l penicillin to prevent bacterial growth. Plates were incubated for 4 days at 25°C in the dark after which Fo outgrowth was assessed.

### Analysis of Fungal Colonization by Quantitative PCR

Tomato roots were harvested 3 weeks after inoculation. Soil was removed from the roots using water, and roots were snap-frozen in liquid nitrogen, followed by overnight freeze-drying. The frozen roots were ground in liquid nitrogen using a mortar. Approximately 100 mg of the resulting powder was used for gDNA isolation and purification using the GeneJET plant Genomic purification Kit (Thermo Scientific). DNA concentration and quality were estimated using a Nanodrop (Thermo Scientific), and by agarose gel electrophoresis. Each 10 μl qPCR reaction contained 10 ng of gDNA template, 10 pM of each primer and 2 μl of HOT FirePolEvaGreen qPCR Mix Plus (Solis BioDyne). The primer sequences for plant tubulin FP2147 (CAGTGAAACTGGAGCTGGAA), FP2148 (TATAGTGGCCACGAGCAAAG) and for Fo IGS FP8498 (TTTGCCATACTATTGAATTTTGC), FP8499 (ACTTTTACCTACCCGGCAGCTC) were used. The cycling program was set to 15 min at 95°C, 40 cycles of 15 s at 95°C, 1 min at 60°C, 30 s at 72°C. The melting curve analysis was performed afterward as follow: 15 s at 95°C, 1 min at 60°C, 15 s at 95°C. For IGS primers two standard curves (four- or ten-times dilution) were performed with a starting concentration of 10 ng resulting with a primer efficiency of 110 and 95%, respectively. Three technical replicates were used per biological sample, and data was normalized to plant tubulin gene level, using qbase + 3.3 (Biogazelle) (Supplementary Figure 2). This experiment was repeated for strains Fo1, Fo3, Fo4, Fo8, Fo10, Fo11, Fo17, and Fo57 and again we were able to detect the Fo strains (Supplementary Figure 2).

### Statistical Analyses

Bioassay data (fresh weight, disease index) was analyzed using a Kruskal–Wallis test using Fo47:Fol4287 as control group and corrected for multiple comparisons using Dunn’s test if not stated otherwise. Comparisons of the number of *mimps*, predicted effectors and core and accessory genome sizes were done using a Mann–Whitney test. All data was analyzed in PRISM 7.0 (GraphPad).

### Whole Genome Sequencing and *de novo* Assembly

*Fusarium* freeze-dried mycelium was harvested from fungus that was grown for 5 days in minimal medium (see above) and used for genomic DNA isolation by multiple phenol:chloroform:isoamylalcohol extractions (25:24:1) followed by DNA precipitation and resuspension in Mili-Q water as previously described (van Dam et al., 2016). The genomes of the other 44 *Fusarium* strains were sequenced by Illumina (150 bp paired-end, insert size ∼450 bp) on a HiSeq 2500 machine by the Hartwig Medical Foundation (Science Park, Amsterdam, Netherlands). Low quality base pairs were filtered and adapter sequences were trimmed with Trimmomatic (parameters: ILLUMINACLIP:TruSeq3-PE-2.fa:2:20:8:4:false SLIDINGWINDOW:4:20 MINLEN:100, where TruSeq3-PE-2.fa is a file with TruSeq adapter sequences that is included in the Trimmomatic package). We used CLC-workbench 8.0 with default settings and minimum contig length 500 to generate *de novo* assemblies (Supplementary Table 1).

### Whole-Genome Alignments

We compiled a dataset of the 44 assemblies we sequenced and assembled for this study, a high-quality SMRT assembly of Fo47 (GenBank Accession GCA_014324445.1) (Li et al., 2020) and 102 other, publicly available Fo genomes we downloaded from GenBank on January 25, 2019 (Supplementary Table 2), arriving at a set of 147 whole-genome assemblies. Of these, 51 were considered endophytes, the 44 strains sequenced for this study, Fo47, well-known as a biocontrol strain, FoMN14 that was isolated from a tomato field but was found to be non-pathogenic on tomato (Gale et al., 2003), and five strains that were isolated from onions but were found to be non-pathogenic on onion (Taylor et al., 2016). To infer all-against-all alignments for the assemblies in this dataset we used *nucmer* with *--maxmatch* and otherwise default settings from the MUMmer package (version 3.23). The *nucmer* output was converted to tabular format using *show-coords* from the MUMmer package without any filtering. For each assembly we identified core or accessory regions as follows: for each whole genome alignment of assembly X with assembly Y_*i*_, we selected aligned regions in assembly X based on percent identity (min. 90%) and length (min. 1 kb), saved them in bed format and merged overlapping regions with *bedtools merge* (Bedtools v2.27.1, default settings) to obtain a set of non-redundant regions in assembly X that were aligned to assembly Y_*i*_. We then used *bedtools genomecov* (with *-bga -split*) to obtain per position in assembly X the number of assemblies Y that aligned to that position (i.e., included that position in at least one alignment) (code available)^1^. Positions that aligned to at least 90% of the other genome assemblies in the dataset were considered core, the rest was considered accessory (Supplementary Figure 3).

### Phylogenetic Analysis

We inferred phylogenetic relations between the 44 endophytes sequenced in this study, Fo47 and 102 other Fo isolates for which whole genome sequences were available on GenBank, and *Fusarium fujikuroi* IMI58289 (GenBank accession GCF_900079805.1). More specifically, we used the BUSCO pipeline (with options ‘-genome –species fusarium_graminearum –blast_single_core –l sordariomyceta_odb9,’ implementing augustus version 3.3.2) to predict and select conserved, single-copy genes in the 147 assemblies in our dataset (Stanke and Waack, 2003; Waterhouse et al., 2018). We used Muscle v3.8.31 to infer multiple sequence alignments for 3003 gene sequences (including introns) that occurred as a single copy in all 148 assemblies, trimAl (v1.4.rev22, with parameter ‘-strict’) to trim the multiple sequence alignments and a custom Python script to concatenate these alignments and create a partition file (Edgar, 2004; Capella-Gutierrez et al., 2009). We used IQ-TREE to infer a phylogeny based on this partition file and concatenated alignment (IQ-TREE version 1.16.12 with parameters ‘-bb 1000 -m GTR -bsam GENESITE’ (Nguyen et al., 2015; Chernomor et al., 2016; Hoang et al., 2018). We visualized the phylogenetic tree using a custom Python script implementing ETE3 (Huerta-Cepas et al., 2016). Branches with bootstrap support below 70% were collapsed.

For phylogenetic analysis of *SIX* homologs, we extracted homologous nucleotide sequences (if present) from 101 publicly available genomes (Fom001 was excluded) (Supplementary Table 1) with BLASTN (with -evalue 0.001, percent identity ≥ 30). We used Clustal Omega to infer a multiple sequence alignment for each query (Sievers and Higgins, 2014). For each alignment a phylogenetic tree was inferred with 100 bootstrap replicates with PhyML 3.0, with HKY85 as the substitution model and a discrete gamma model with four categories (Guindon et al., 2010).

### Putative Effector Gene Identification and Hierarchical Clustering

*De novo* prediction of candidate effector genes from the 45 Fo endophytic genomes (incl. Fo47) was performed as described in van Dam et al. (2016) (*e*-value 0.001, percent identity > 60%, alignment length > 60%) using the custom Python scripts from https://github.com/pvdam3/FoEC. After *de novo* prediction, all 157 ORFs identified by the pipeline were compared with the 104 putative effectors previously described (van Dam et al., 2016) to remove redundant ORFs. Among these ORFs, homologs of the previously described putative effector genes (defined as *e*-value of 1*e*-03; percent identity of >60%; alignment length of >60%) were removed from the list. After this selection, 135 unique ORF were identified and their signal peptide (SP) was analyzed by a custom Python script^2^, because we noticed after visual inspection that SignalP (employed in the method of van Dam et al., 2016) returns some unlikely SPs. In short, a true SP was defined as an N-terminal region of 15–30 amino acids containing a stretch of nine to 20 consecutive mostly hydrophobic residues that starts at least after the first and at most after the eighth residue, and ends at most two residues before the last three amino acids. Specifically, this stretch contains no glutamine, asparagine or charged (acidic or basic) residues and a maximum of three hydroxylated residues. The last three residues of the signal peptide consist of a small amino acid (G, A, V, S, T, or C), any amino acid followed by another small amino acid. A total of 81 ORFs met these SP selection criteria. From these, we selected those that encode mature proteins longer than 34 amino acids, resulting in 66 new candidate effectors (Supplementary Table 4). These, together with the 104 previously published candidate effectors, were used for further analysis. For hierarchical clustering, the script of van Dam et al. (2016), was slightly modified to highlight secreted enzymes (defined by the presence of “ase” in the name) and location of effectors candidates located on the core or accessory genome in Fo47. The script, together with the input file that contains 170 putative effectors and three conserved genes: *EF1-alpha*, *RPB1*, and *RPB2* can be found on https://github.com/marads/FoEC.

### Data Access

The Whole-Genome Shotgun projects for the 44 newly sequenced strains have been deposited at GenBank under the Bioproject PRNJA587975. Raw sequence data has been deposited into the Sequence Read Archive under accession numbers SRX7263439 and SRX7124613 - SRX7124656. The other genome sequences used for analyses presented in this paper were retrieved from GenBank and are listed in Supplementary Table 2.

## Results

### Most Endophytes Can Colonize Stems of Tomato Plants Without Causing Disease Symptoms and Confer Resistance Against Fusarium Wilt

We selected 44 Fo strains obtained from soils throughout Australia (Rocha et al., 2016) and asymptomatic plants: red fescue roots from Spain (Pereira et al., 2019) and tomato plants from the United States (Demers et al., 2015). We also included Fo47, a known biocontrol strain (Lemanceau and Alabouvette, 1991; Table 1). To assess the endophytic potential of this set of strains, and to see whether this potential differs between strains isolated from the same plant species and those isolated from other plant species, we tested the 40-spore-producing Fo strains for their ability to colonize tomato seedlings. Most of the Fo strains tested did not cause disease symptoms although plants inoculated with either Fo54 or Fo59, isolated from tomato plants in the United States, occasionally showed minor vessel browning (data not shown). All strains could be re-isolated from asymptomatic tomato stems (Table 1). To confirm plant colonization by Fo, DNA was isolated from roots. Primers for the intergenic-spacer region (IGS), which is multi-copy and is conserved across Fo strains, were used to enhance the sensitivity of the qPCR assay. The colonization level in tomato roots inoculated with Fo47 was found to vary among different experiments and for many strains we observed extensive variation between technical replicates (Supplementary Figures 1a–c). Nevertheless, we observed colonization by all Fo strains in all experiments. We conclude that the selected Fo strains can colonize tomato plants, moving beyond the root system into the stems, yet do generally not cause disease symptoms.

**FIGURE 1:**
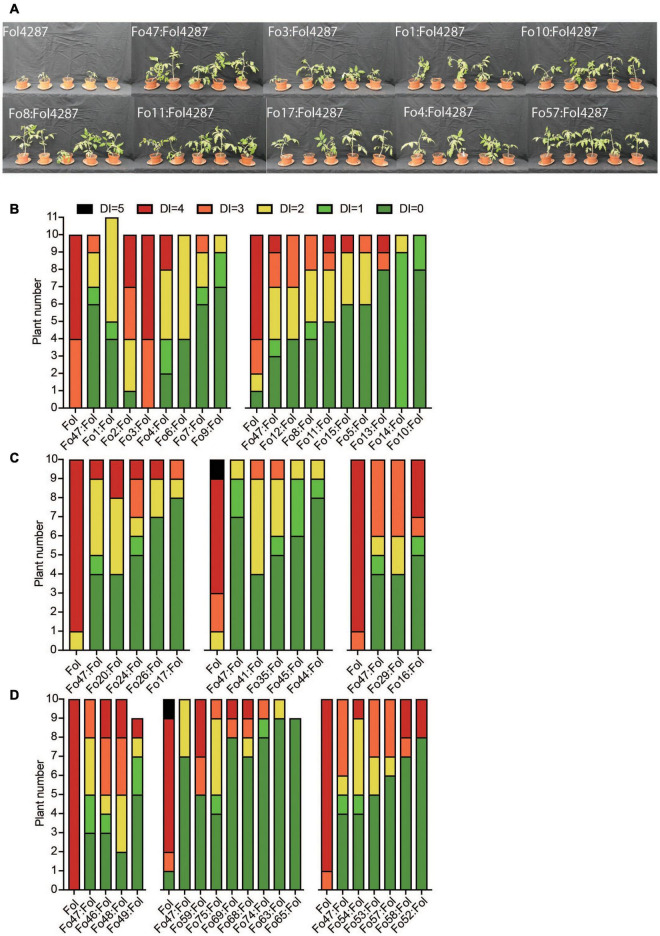
Most Fo strains trigger Fusarium wilt resistance in tomato to a similar level as Fo47. **(A)** Pictures of representative tomato plants 3 weeks after inoculation with Fol4287 alone or together with the endophytic strain Fo3, Fo1, Fo4, Fo10, Fo8, Fo11, Fo17, or Fo57. Most Fo strains trigger resistance against Fusarium wilt in tomato to a similar level as Fo47. Disease symptoms of tomato plants inoculated with Fol4287 or co-inoculated with Fo strains from **(B)** Australian soils, **(C)** grass roots from Spain or **(D)** tomato plants from the United States. Data were analyzed by Kruskal–Wallis test using Fo47:Fol4287 treatment as control group and corrected for multiple comparisons using Dunn’s test.

Next, the ability of 41 Fo strains to confer resistance against Fusarium wilt disease in tomato was determined by scoring disease symptoms following co-inoculation of the Fo strain with the pathogenic Fo4287 isolate. Figure 1A shows a representative set of plants showing suppression of disease symptoms by the Fo endophytes. Among all bioassays Fo47 reduced disease symptom severity, although the extent varied between experiments (Figures 1B–D). A statistical Kruskal–Wallis test revealed that out of the 41 Fo strains only two strains, Fo2 and Fo3, conferred less Fusarium wilt suppression than Fo47 (*P < 0.05, ^***^P < 0.0001). All endophytic Fo strains isolated from grass roots (Figure 1C) and from tomato plants (Figure 1D) conferred resistance to a level similar as Fo47. Taken all together the majority of Fo isolates (38/40) from a diverse collection, colonize tomato plants and suppress Fusarium wilt to an extent similar to the well-characterized biocontrol strain Fo47.

### Endophytism Is Polyphyletic Within the *Fusarium oxysporum* Species Complex

To identify genetic factors that underlie an endophytic lifestyle, we sequenced the genomes of all 44 strains using paired-end Illumina sequencing and achieved 60- to 140-fold coverage (Supplementary Table 1). This resulted in assemblies of 501 (Fo9) to 2857 (Fo3) scaffolds. Assembly size is similar among endophytic strains with Fo2 having the smallest (48 Mb) and Fo3 the biggest (60 Mb) assembly size. BUSCO completeness is also similar among isolates (Supplementary Table 1), and comparable with other Fo genomes deposited in GenBank (Supplementary Table 2).

**FIGURE 2:**
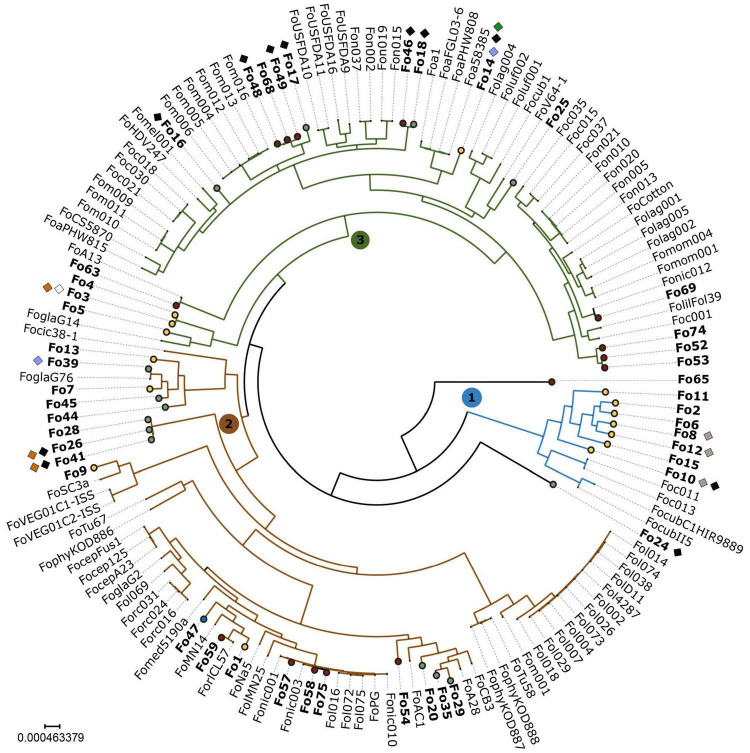
Endophytic Fo have a polyphyletic origin with an unequal distribution among the three clades. In this maximum-likelihood phylogeny based on a concatenated alignment of 3003 conserved genes, three major clades representing different phylogenetic species, are numbered 1, 2, 3 and colored in blue, green, and red. Endophytic strains sequenced for this study are shown in bold and represented with circles. Circles are color-coded according to habitat: yellow – Australia; green – Spain; maroon – United States; blue – France (reference endophytic strain Fo47). Endophytes are polyphyletic: all three major clades contain endophytes. Endophytes from different habitats cluster together (e.g., Fo46 and Fo18), and endophytes cluster with pathogens (e.g., Fo59 with ForlCL57). Presence of known effector genes is indicated with diamonds and colored as follows: white – *SIX1*; gray – *SIX2*; blue – *SIX6*; black – *SIX9*; orange – *SIX14*; and green – *AVRFOM2*. *Fusarium fujikuroi* IMI58289 was used as an outgroup to root the tree and then removed.

To determine phylogenetic distribution of endophytism within the Fo species complex, we used a dataset of 102 publicly available genomes of mainly pathogenic Fo (Supplementary Table 2), the SMRT assembly of Fo47 and the 44 endophyte whole-genome sequences generated in this study. We inferred a phylogeny based on the concatenated alignment of 3003 genes from Fo47 that are present as a single copy gene in all genomes in our dataset (Figure 2). As observed before (O’Donnell et al., 1998; Baayen et al., 2000; Lievens et al., 2007; van Dam et al., 2016), the phylogenetic tree has three major clades, of which at least one clade (clade 1 in blue, Figure 2) corresponds to a separate phylogenetic (cryptic) species (Laurence et al., 2014). Endophytic Fo strains are colored based on location [yellow: Australia, green: Spain, maroon: United States, blue: France (Fo47)] and are dispersed among all three clades (Figure 2). The distribution of endophytic strains among the clades appears, however, uneven. Clade 1 contains only strains isolated from Australia, clade 2 contains mainly strains from the United States and clade 3 contains the majority of strains isolated from Spain. Still, all three habitats contain strains from at least two of the three main clades. Interestingly, two endophytic strains, Fo65 and Fo11, fall outside these three clades, raising the possibility of hybridization or of a new phylogenetic species. Endophytic strains that cluster together originate either from the same location (such as Fo10, Fo15, Fo8, Fo12, Fo6, Fo2, and Fo11), or from different locations such as Fo16, Fo46, and Fo18 (Figure 2).

### Endophytic *Fusarium oxysporum* Strains Have Similar Amount of Accessory DNA as Pathogenic Strains

We expected that the core genome in Fo is very similar between pathogenic and endophytic strains and that genetic factors that differentiate between the two lifestyles would be encoded on the accessory genome. To identify core and accessory chromosomes, we compiled a dataset of the 44 newly sequenced genomes, the SMRT assembly of Fo47, and a set of 102 publicly available Fo whole-genome sequences. We defined core regions as genomic regions that align to more than 90% of the assemblies in our dataset and designated the rest of the genome ‘accessory’ (see Materials and Methods for more detail and Supplementary Figure 3 for an example based on the assembly of Fo47). Having thus defined core and accessory regions in all genomes in our dataset, we could compare the size of the core and accessory regions between pathogenic strains and endophytes. For comparability, strains sequenced with SMRT sequencing (including Fo47) for which we expected a more complete assembly of repetitive regions and thus a larger accessory genome, were omitted from this analysis.

**FIGURE 3:**
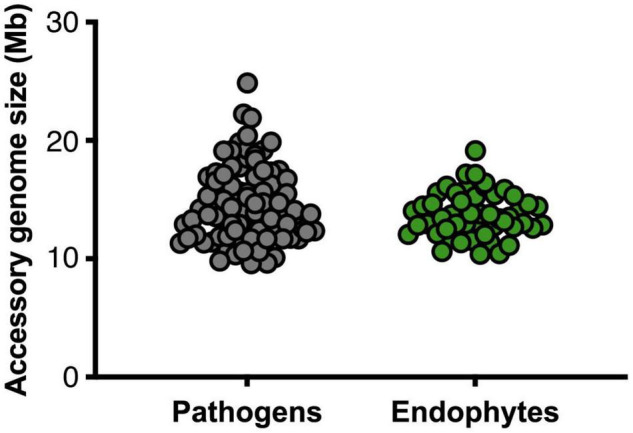
Pathogens do not have larger accessory genomes than endophytes. The sizes of the accessory genomes – i.e., genomic regions that are present in less than 90% of the other 147 Fo genomes in the dataset – in million base pairs for pathogens (gray, left) and endophytes (green, right) (*p*-value 0.16, non-parametric Mann–Whitney *U* test). Genome assemblies obtained by SMRT sequencing were excluded from the comparisons of accessory genome sizes.

The size of the core and accessory regions is similar among endophytic isolates - most having a core genome size of around 37.7 Mb (Supplementary Table 1) – with Fo2 having the smallest (10 Mb) and Fo3 the largest amount (19 Mb) of accessory DNA (Supplementary Table 1). Similarly, the size of the accessory genomes of pathogenic strains ranged from 9 Mb (FocubII5) to 24 Mb (FoaFGL03-6) (Supplementary Table 2). Overall, therefore, Fo endophytic strains have a similar core and accessory genome size as pathogenic strains (Figure 3). Among pathogenic strains, however, a few exceptions were observed (Figure 3). Foc037 and Foa1 have a smaller amount of core DNA while FoaFGL03-6 is an outlier that appears to have more accessory material than the other strains.

### Endophytism Is Not Associated With Specific Accessory Sequences

**FIGURE 4:**
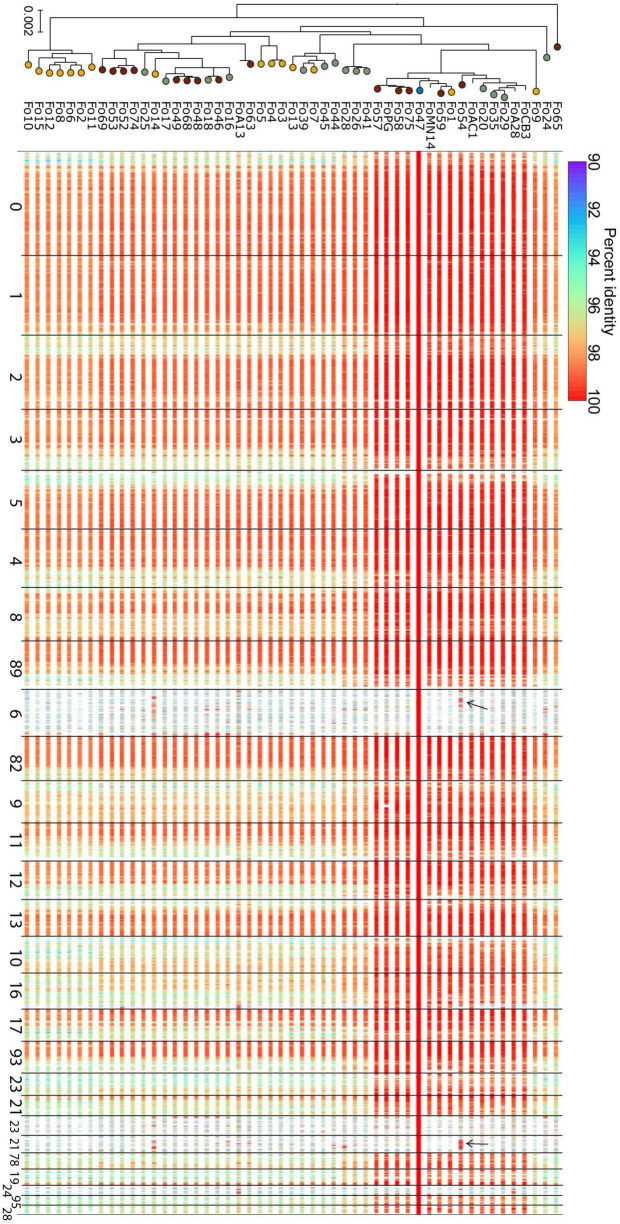
Comparative genomics does not reveal any endophyte-specific genomic regions. Each row depicts the aligned regions that span more than 1 kb in the reference genome and are more than 90% identical in sequence, from a whole-genome alignment between Fo47 and one of the endophyte Fo genomes (incl. Fo47 itself) in our dataset. Rows are ordered according to position in the phylogenetic tree (left, this is the same tree as in Figure 1, except for the fact that all pathogenic isolates have been removed). Lines are color-coded according to percent identity (see scale on top of the panel), where red lines represent 100% identical sequences. Most contigs are highly conserved among different Fo strains: these are considered core contigs. In contrast, large accessory contigs, such as contig 6, 21, 78, and 95 are less conserved (see also Supplementary Figure 2). Few regions of Fo47 accessory material are shared among other isolates, for example, part of contig 6 and contig 78 is shared with Fo54 (indicated with black arrows). The high level of sequence identity of these shared accessory regions, compared to the level of sequence identity of core regions, suggests these regions were horizontally transferred between the two Fo lineages.

Strains of Fo that are pathogenic on the same host often share a “pathogenicity chromosome” that confers pathogenicity toward that host, for instance in Fol4287 this is chromosome 14 (Ma et al., 2010; van Dam et al., 2017; Li et al., 2020). Accessory chromosomes are in general less conserved than core chromosomes, yet pathogenicity chromosomes are typically highly conserved between strains that cause disease to the same host. We asked whether we could identify an endophyte-specific -chromosome or -chromosomal region and compared sequence similarities of alignments with the core and accessory genome of Fo47 to the genomes of other endophytes. Figure 4 shows that Fo47 core regions are indeed shared among all strains with high sequence similarity (>96% identical, except for subtelomeric regions). The Fo47 assembly has four major (>400 kb) accessory contigs, contigs 6, 21, 78 and 95, but these are not generally shared with the other endophytes in our dataset (see also Supplementary Figure 3). One notable exception is Fo54 that shares a small part of the accessory genome of Fo47, contig 78 and part of contig 6, with almost 100% identity. Overall, we conclude there is not a specific accessory region that is shared among all or most Fo endophytes.

### Prediction of Effectors in 45 Endophytic *Fusarium oxysporum* Genomes Yields 66 New Effector Candidates

**FIGURE 5:**
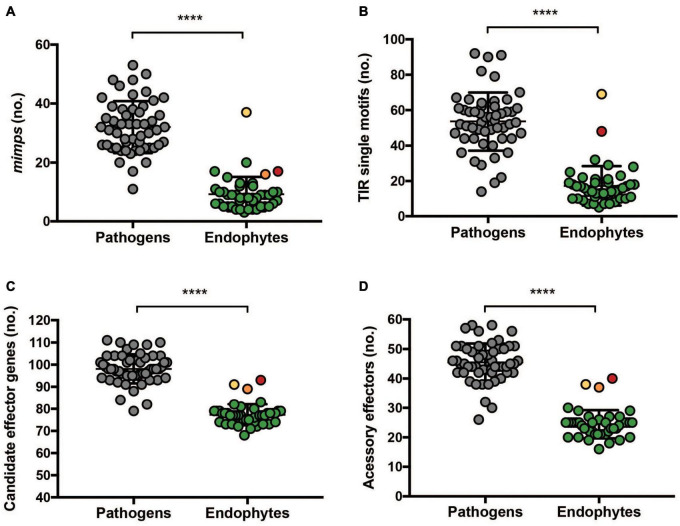
Endophytic Fo have fewer *mimps* and fewer putative effectors than pathogenic Fo. **(A)** Complete and **(B)** incomplete terminal inverted repeats (TIR) were predicted from 45 endophytic genomes and 53 pathogenic isolates (ForlCL57, FOSC3A, and FoMN14 were omitted from the analysis for not being pathogenic nor tested for endophytism on tomato and Fol4287, Forc016, and Fo47 were omitted because they were sequenced by Pacbio). Data were analyzed by a non-parametric Mann–Whitney *U* test (****P < 0.0001). Endophytic isolates (45 strains) and 55 pathogenic strains were used for predicting **(C)** candidate effectors and **(D)** candidate effectors located on the accessory genome. Accessory effectors were defined as candidate effectors which do not have a homolog on the core genome of Fo47. The colors correspond to Fo14 (red), Fo10 (orange), and Fo3 (yellow). Data were analyzed by a non-parametric Mann–Whitney *U* test (****P < 0.0001).

Next, we considered the possibility that endophytism could require a specific set of effectors, possibly distributed among different core or accessory chromosomes. To test this hypothesis, the 45 endophytic Fo genomes were used to predict *de novo* putative effector genes based on the *mimp* TIR identification motif as previously described (van Dam et al., 2016). Endophytic genomes have fewer *mimps* than pathogenic strains (Supplementary Table 3 and Figures 5A,B) with Fo3 having the highest number of *mimp* TIR motifs (Supplementary Table 3). Due to the low presence of *mimps* only 157 ORFs close to a *mimp* and potentially encoding secreted proteins were identified. To remove redundancy, effector candidates identified previously (van Dam et al., 2016) were filtered out using a self-BLAST. The sequences of the remaining putative effectors were analyzed for the presence of a *bona fide* signal peptide using a refined prediction tool described in Materials and Methods. The 66 remaining newly identified putative effectors, having a predicted mature protein length of at least 35 amino acids, are summarized in Supplementary Table 4.

### Endophytic Strains Cluster Together Based on Scarcity of Effector Candidate Genes

**FIGURE 6:**
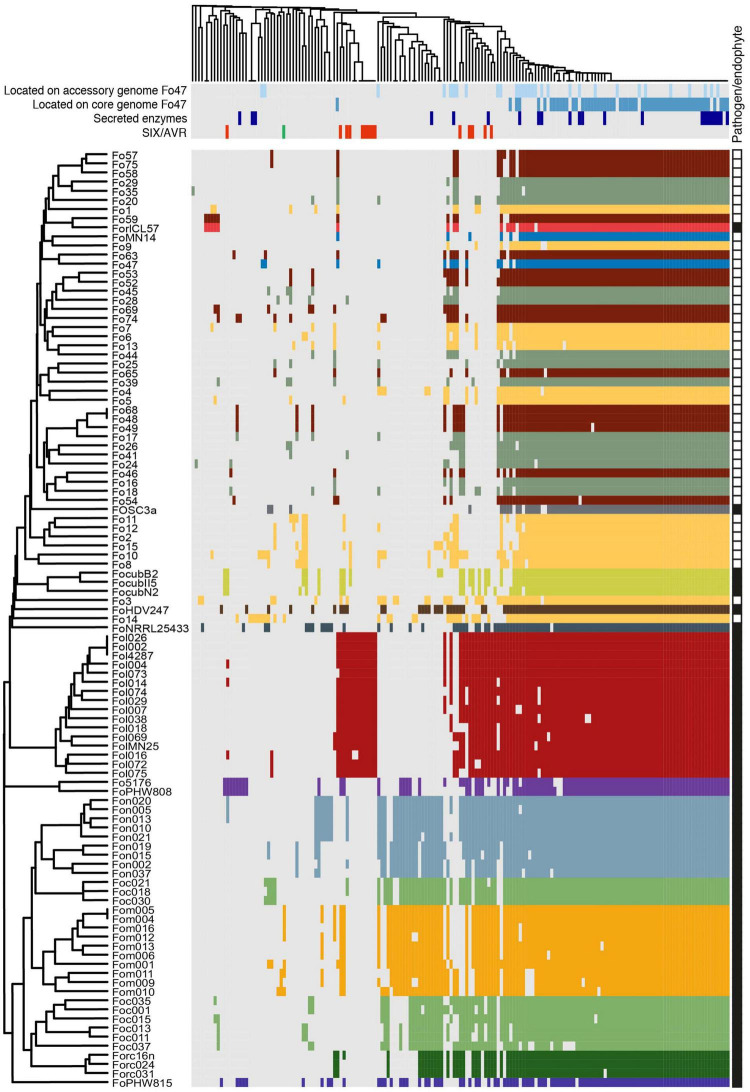
Most endophytic Fo cluster based on presence/absence of 170 candidate effectors genes from 103 genomes. Gray represents absence, while color represents presence of the candidate effector genes. Strains with the same host-preference (pathogens) or from the same habitat (endophytes) have the same color. For the endophytes we followed the same color scheme as in Figure 1: yellow – Australia; green – Spain; maroon – United States, blue – France (Fo47). FoMN14 was not sequenced for this study, but is an endophyte isolated from tomato plants in the United States and therefore colored maroon. Whether strains were considered ‘pathogen’ or ‘endophyte’ is indicated with black and white squares, respectively, in the rightmost column. On top, light blue: candidate effector genes which are located on the accessory genome of Fo47; dark blue: candidate effector genes which are located on the core genome of Fo47; dark purple: putative secreted enzymes; red: *SIX* genes, green: *AVRFOM2*. The newly identified putative effectors are summarized in detail in Supplementary Table 5.

To determine the effector repertoires of endophytic Fo strains, and compare these to those of pathogenic Fo strains, presence/absence of candidate effector candidates was determined for 103 genomes of pathogenic and endophytic Fo strains. Newly identified candidate effectors from the endophytic genomes were combined with the 104 candidate effectors previously identified from pathogenic strains (van Dam et al., 2016). The presence of genes for these 170 candidate effectors in the 103 genomes was determined by blastn using a threshold percentage (number of identical nucleotides/query length) of ≥30%. Notably, hierarchical clustering of presence/absence patterns of effectors largely divided pathogenic from endophytic Fo strains (Figure 6): 43 out of 45 endophytes cluster together and this cluster contains only two reported pathogens: ForlCL57, causing root rot on tomato, and FOSC3a, a clinical isolate. Notably, endophytic strains did not cluster together based on the source they were isolated from (grass roots, tomato plants or the Australian continent). This contrasts with pathogenic strains that cluster together based on their host specificity (Figure 6) as previously observed (van Dam et al., 2016). Interestingly, the endophytic isolates Fo3 and Fo14 fall outside the ‘endophytic’ cluster (Figure 6). These two strains also have a relatively high number of *mimps* and/or candidate effector genes (Figure 5) compared to the other endophytic strains (Supplementary Table 3).

The clustering of endophytes appears to be based on the scarcity of effector candidates in endophyte genomes rather than the presence of specific “endophyte effectors.” Statistical analysis on predicted number of candidate effector genes in pathogens and endophytes supports this hypothesis (Figures 5C,D). To define the localization of candidate effector genes in either core or accessory genome, the genome of Fo47 was used as a reference after defining its core and accessory regions as described in Materials and Methods. A blastn search of all 71 candidate effector genes of Fo47 was performed against either its core or accessory regions. Candidate effector that gave a blast hit on core contigs were designated “core effectors” (represented in dark blue in Figure 6), while the ones mapping on the accessory genome were defined as “accessory effectors” (colored in light blue) (Figure 6). Out of 78 effector genes, 55 were located exclusively on the core genome (70.5%), 16 on the accessory genome (20.5%) and 7 had homologs on both accessory and core genomes (9%). As expected, candidate effector genes shared among (almost) all Fo strains are localized on the core genome and this category appears to be enriched for secreted enzymes (purple) (Figure 6). Therefore, the difference in the number of candidate effector genes between pathogenic and endophytic strains is not due to the number of effectors located on the core genome but rather due to a larger number of candidate effector genes located on the accessory genome of pathogens (Figures 5C,D).

### Sequence Comparison of *SIX* Gene Homologs Reveals Possible Horizontal Gene Transfer

In line with having a smaller number of candidate effectors, endophytic Fo strains have few *SIX* gene homologs (Supplementary Table 4). Out of 14 *SIX* genes identified in Fol, only homologs of *SIX1*, *SIX2*, *SIX6*, *SIX9*, and *SIX14* are sporadically present in endophytic strains (Supplementary Table 4). The Australian collection of strains seems to be the most diverse since it has homologs of all five genes, while the Spanish collection of grass root endophytes contains only homologs of three genes (*SIX6*, *SIX9*, and *SIX14*), and the endophyte collection from tomato plants in the United States has merely *SIX9* homologs.

**FIGURE 7:**
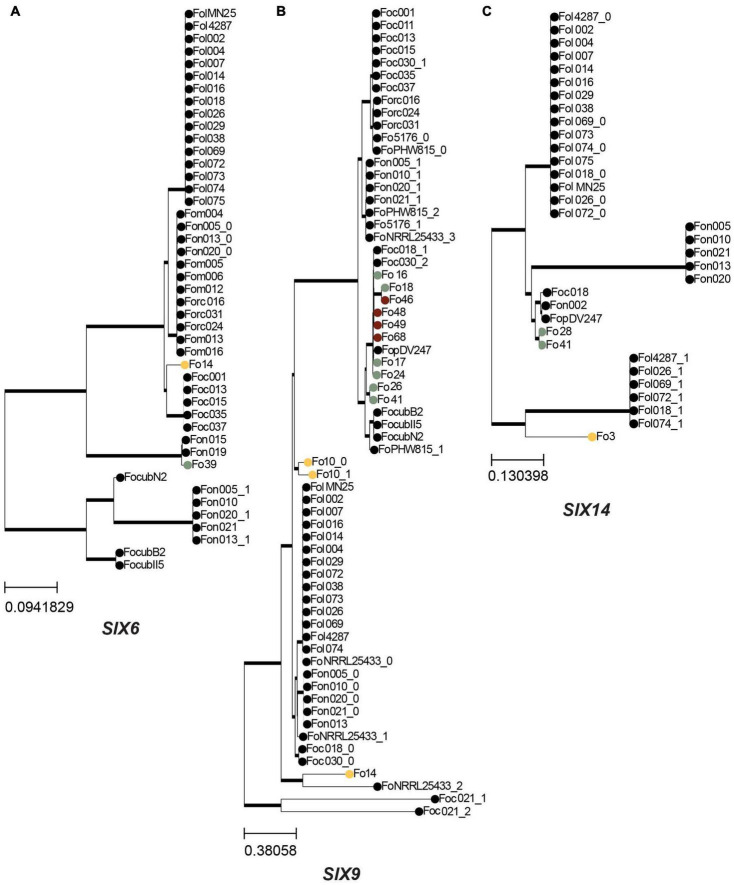
*SIX* genes are generally vertically inherited in endophytic isolates. A maximum-likelihood phylogeny was inferred based on a multiple sequence alignment of the nucleotide sequences of *SIX* genes that are present in endophytes. Trees were rooted with midpoint rooting, thick lines indicate branches with 100% bootstrap support, colored circles indicate endophytes sequenced in this study, following the same color scheme as in Figure 1: yellow – Australia; green – Spain; maroon – United States, other Fo isolates are shown as dots. **(A)** Phylogeny for *SIX6*. **(B)** Phylogeny for *SIX9*. **(C)** Phylogeny for *SIX14*.

To determine the relationship between *SIX* homologs from endophytic strains to those of pathogenic strains, their sequences were aligned and phylogenetic trees were inferred. Moreover, using the output of the candidate effector prediction pipeline, we determined whether a *mimp* was detected upstream of each *SIX* homolog. *SIX1* and *SIX2* homologs were found exclusively among Australian strains (Supplementary Table 4). Fo15 and Fo8 have an identical *SIX2* gene sequence that is highly similar to the *SIX2* homolog in Fo10 and two Fo f. sp. *cubense* strains (data not shown). Two endophytic strains have a *SIX6* homolog (Fo14 and Fo39). The Fo39 strain has a *SIX6* homolog identical to that of Fo f. sp. *niveum* strains Fon15 and Fon19 (Figure 7A). This is remarkable since in the core genome phylogenetic tree these *Fon* strains cluster together on clade 2 while Fo39 belongs to clade 3 (Figure 2), suggesting the possibility of horizontal transfer of *SIX6*. The most common *SIX* gene among endophytic strains is *SIX9* (12/45 strains). *SIX9* has a phylogeny mostly congruent with the core genome (Figure 7B), suggesting vertical inheritance combined with gene loss. However, an exception is Fo17 and Fo24 that have identical *SIX9* sequences but are phylogenetically distant, suggestive of another horizontal gene transfer event. In the case of *SIX14* (Figure 7C), two Spanish strains (Fo28 and Fo41) have identical sequences, while the *SIX14* homolog in Fo3 (Australia) is more closely related to Fol *SIX14*.

Overall, we conclude that the *SIX* gene sequences present in endophytic strains mostly follow the same phylogeny as the conserved core genes described in Figure 2. However, there are a few indications for horizontal gene transfer of *SIX6* and *SIX9* homologs.

## Discussion

*Fusarium oxysporum* is among the most frequently isolated fungal species from roots of healthy plants (Inami et al., 2014; Pereira et al., 2019). Clearly, endophytism is common among Fo, but it was unclear if this lifestyle (endophytism versus pathogenicity) is genetically determined or simply depends on the presence of a susceptible host. By using comparative genomics, we conclude that genomes of pathogenic Fo strains differ in quantitative aspects from genomes of endophytic Fo strains. Furthermore, the observation that the majority of tested Fo isolates (38/40) suppresses Fusarium wilt to an extent similar as the well-characterized biocontrol strain Fo47, indicates that endophytism and disease suppressing ability is a common trait among Fo in tomato. The latter is surprising as previous studies found more variation in biocontrol potential among non-pathogenic Fo strains. For example, Schneider found that Fo strains from a disease-conducive soil did not prevent Fusarium wilt of celery as well as Fo strains from a Fusarium wilt-suppressive soil (Schneider, 1984). Likewise, only about half of the 118 Fo strains isolated from a Fusarium wilt-suppressive soil reduced Fusarium wilt of tomato (Larkin et al., 1996). It will be interesting to monitor the biocontrol potential of the here described Fo strains on other tomato varieties or plant species to test whether the observed effect could be tomato cultivar specific and/or is due to the inoculation method used. The root-dip inoculation method in which roots are wounded might make seedlings more susceptible to endophytic colonization than plants encountering Fo propagules *in situ*, possibly resulting in a potentiated protection against disease-causing Fo strains.

*De novo* prediction of candidate effectors yielded fewer candidates from endophyte genomes than from pathogenic strains (van Dam et al., 2016). Since our effector gene prediction method was based on the proximity of *mimp* TIR sequences to an ORF, this lower number can be at least partially attributed to the fact that endophytes have less of these miniature transposons (Figures 5A,B). Potential effector candidates that are not in conjunction with *mimps* will not be identified by the prediction method. Since the Fo core genome is highly conserved, and transfer of single pathogenicity chromosomes suffices to turn an endophyte into a pathogen (Ma et al., 2010; Vlaardingerbroek et al., 2016; van Dam et al., 2017), it is unlikely that core genome-encoded genes are responsible for the difference between endophytic and pathogenic lifestyles. In correspondence, comparative genomics between the root endophyte *Colletotrichum tofieldiae* and the closely related pathogenic *Colletotrichum incanum* showed that the endophytic strain encodes fewer putative effector proteins (Hacquard et al., 2016). Taken together, it seems that a pathogenic ‘lifestyle’ favors (or requires) an extended effector gene catalog.

Candidate effectors unique to, or enriched in, endophytic strains (as compared to pathogenic strains) were not identified. This finding supports the hypothesis that all Fo strains are potentially endophytic and that pathogenic strains can be endophytes in non-host plants. Since we did not observe candidate effector genes on the accessory genome unique to endophytic strains isolated from the same plant species, it is likely that host-specific effectors do not play an important role in host- adaptation of endophytic stains, if such host-specific adaptation occurs at all (Demers et al., 2015). However, considering that many candidate effector genes are conserved within Fo and are located on the core genome, it could be that (some of) these ‘core’ effectors facilitate root endophytism. This would explain why Fo is such a “promiscuous” root endophyte, and why pathogenic strains behave as endophytes in a non-host plant (Gordon et al., 1989), while pathogenic behavior is exceptional and conferred by an additional set of “host-specific” effectors.

Among Fo effectors, SIX proteins, identified as proteins secreted in the xylem of Fol-infected tomato plants (Houterman et al., 2007), have been shown to contribute to aggressiveness in various ff. spp. such as *lycopersici*, *cubense*, and *melonis* (van Dam et al., 2017; Widinugraheni et al., 2018). Interestingly, *SIX9* homologs are the most common *SIX* gene homologs among the endophytic strains analyzed in this study. An earlier survey of Fo strains from Australian soils also identified *SIX9* as the most abundant and conserved *SIX* gene (Rocha et al., 2016). Currently, the function of the encoded protein is unknown, but it does not seem to contribute significantly to pathogenicity in Fom, nor in Fol (Vlaardingerbroek et al., 2016; van Dam et al., 2017). Host colonization by endophytic Fo strains, such as Fo47, is typically restricted to the root cortex (Benhamou and Garand, 2001), and it is unclear whether *SIX* homologs are expressed in endophytic strains. In any case, it seems unlikely that these *SIX* homologs have a role in endophytic root colonization, since endophytic strains without *SIX* gene homologs (such as Fo47) can reach tomato stems equally well as strains with one, two or three *SIX* gene homologs.

Our clustering method, based on candidate effector genes, separates almost all pathogenic Fo strains from the other, presumably exclusively endophytic strains. Since Fo endophytes are characterized by scarcity of *mimps* and accessory candidate effectors we expect that it is possible to predict ‘lifestyle’ based on whole genome sequences. Of course, bioinformatics pipelines cannot fully substitute experimental validation of strains’ lifestyle, but likely non-pathogenic strains can be predicted with a high confidence based on scarcity of effector candidates. It is important to note, however, that predicting endophytic strains based on candidate effector genes can lead to false negatives: strains predicted to be pathogenic could still be avirulent, for instance because they fail to express effector genes or have other ‘defects’ (Jelinski et al., 2017).

The accessory genome of Fo has been shown to be dispensable (i.e., not required for Fo viability or metabolic versatility) but it contributes to pathogenicity toward a specific host (Ma et al., 2010; Vlaardingerbroek et al., 2016; van Dam et al., 2017). By comparing the genomes of Fo endophytes with pathogens, we observed that they have similar genome sizes, but endophytes have fewer *mimps* and accessory effector candidates. It is unclear which functions, if any, the still sizable (10 to 20 Mbp) accessory genomic regions have in Fo endophytes. These regions may harbor genes necessary for interaction with other (micro)organisms and/or could serve as genomic ‘playground’ for evolution of new traits.

We estimated genome size based on assemblies generated with short sequencing reads, which may have led to an underestimation of true genome size due to ‘collapse’ of multiple copies of identical sequences. This is true for both endophyte and pathogen genomes, but if one of these groups has more active or recently active transposons there may a difference in the size of the accessory genome that we can only observe using data from long-read sequencing.

The 45 Fo endophytes analyzed are polyphyletic within the Fo species complex, with an apparent unequal distribution (based on location) of endophytic strains among the three clades previously described (O’Donnell et al., 1998; Lievens et al., 2007; van Dam et al., 2016). A polyphyletic origin of pathogenic and endophytic isolates corresponds with previous observations (Inami et al., 2014; van Dam et al., 2016), and confirms the notion that pathogenicity evolved multiple times during Fo evolution from a largely endophytic population.

Here, we assumed that strains isolated from asymptomatic plants or from soil, in contrast to strains isolated from diseased/symptomatic plants, predominantly have an endophytic lifestyle. However, as we mentioned above, it may be the case that some of these strains are pathogens on an unidentified host. When comparing the three endophytic ‘populations’ investigated here, the Australian strains seem to be the most diverse in terms of effector number with Fo3 and Fo14 being outliers, suggesting perhaps a larger “pathogenic potential.” The higher diversity among the Australian population could be related to sampling strategy: samples were collected from across an entire continent while the other two populations were collected from smaller areas. Moreover, the Australian isolates were collected from soil and may thus include strains with a more saprotrophic lifestyle, while the other two populations were isolated from within plants (tomato or red fescue).

Concluding, we propose that an endophytic lifestyle is common among Fo strains, possibly requiring a set of core effectors, while pathogenicity is an exception and determined by a specific set of effector genes expressed upon interaction with a susceptible host. In general, endophytic lifestyles seem to be typical among soil-borne microorganisms, such as *Verticillium* and *Pseudomonas*, which are traditionally studied as plant pathogens (Malcolm et al., 2013; Melnyk et al., 2019). Importantly, these endophytes can reduce host susceptibility to pathogenic strains of *Fusarium*, *Verticillium*, and *Pseudomonas* (Fravel et al., 2003; Grosskinsky et al., 2016; Deketelaere et al., 2017; de Lamo and Takken, 2020). Pathogenicity and biocontrol appear to co-exist within many species. To apply endophytes for disease control, it is of the utmost importance to be able to deduce from the genome sequence whether a strain used for biocontrol could potentially be pathogenic in interactions with certain plant species. We show that this is possible for *Fusarium oxysporum*.

## Data Availability Statement

The original contributions presented in the study are publicly available. This data can be found here: National Center for Biotechnology Information (NCBI) BioProject database under accession number PRNJA587975.

## Author Contributions

MC performed gDNA isolations of the endophytic strains, carried out bioassays to test for their pathogenicity and performed fungal re-isolation assays. LF assembled the genomes and inferred the phylogenetic tree in Figure 1. MS developed a script to identify signal peptide from fasta files and modified the effector prediction pipeline to distinguish core and accessory candidate effectors. MC and MS predicted and clustered effectors and inferred phylogenetic trees for predicted effectors. MC, MS, LF, FT, and MR designed the experiments and critically revised the manuscript. LF and MC analyzed the whole-genome sequences. MC, LF, FT, and MR wrote the manuscript. All authors contributed to the article and approved the submitted version.

## Conflict of Interest

The authors declare that the research was conducted in the absence of any commercial or financial relationships that could be construed as a potential conflict of interest.

## Publisher’s Note

All claims expressed in this article are solely those of the authors and do not necessarily represent those of their affiliated organizations, or those of the publisher, the editors and the reviewers. Any product that may be evaluated in this article, or claim that may be made by its manufacturer, is not guaranteed or endorsed by the publisher.

## Footnotes

1
https://github.com/LikeFokkens/Classify_Core-Accessory

2
https://github.com/marads/signal_peptide_scripts

## Supplementary Material

Supplementary Figure 1 Fo3 colonizes tomato roots poorly and does not protect against Fusarium wilt disease. **(a)** Pictures of representative tomato plants 3 weeks after inoculation with Fol4287 only or together with the endophytic strain Fo3, Fo1, Fo4, Fo10, Fo8, Fo11, Fo17, or Fo57. Fresh weigh above cotyledon level **(b)** and disease symptoms **(c)** were scored 3 weeks after inoculation. Fresh weight data were analyzed using Kruskal–Wallis test with a Dunn’s multiple comparisons test where Fol4287 was defined as control group.

Supplementary Figure 2 All Fo strains colonize tomato roots. Relative quantification of Fo strains isolated from **(a)** Australian soils **(b)** grass roots from Spain or **(c)** tomato plants from the United States with respect to plant tubulin. **(d)** Repetition of the experiment with selected strains.

Supplementary Figure 3 Core and accessory regions in Fo47. Whole-genome alignments were inferred for Fo47 and the other 146 Fo genomes in our dataset. This plot shows for each position on the Fo47 genome, the number of other Fo genomes this position aligns with (including only alignments that span more than 1 kb and are more than 90% identical). Positions that align with more than 90% of the other Fo genomes (i.e., at least 133 other genomes, threshold indicated with top blue line) are considered ‘core.’ Core regions that span more than 10 kb are shown in blue at the bottom. Contig 6, 21, 78, 95 and several very small contigs are largely considered accessory. Contigs are ordered according to size. This approach was used for all other genomes in our dataset, allowing us to compare sizes of core and accessory genomes of assemblies achieved through similar sequencing strategies (Figure 1).

Supplementary Table 1 Statistics of *de novo* genome assemblies generated in this study.

Supplementary Table 2 Overview of the genome assemblies collected from GenBank and used in this study. All genomes were used for phylogenetic analysis and determining core/accessory material unless otherwise stated.

Supplementary Table 3 Overview of *mimps* (single and complete TIR motifs) and number of candidate effector genes (located on the core and accessory regions) among endophytic and pathogenic isolates used for the hierarchical clustering.

Supplementary Table 4 Distribution of SIX genes and MAT type among endophytic strains. Effector candidate genes present in the endophyte genomes are highlighted in yellow. Presence of a *mimp* in proximity to their open reading frame is outlined by red borders.

Supplementary Table 5 Effector candidates newly identified from 45 endophyte genomes.

## Abbreviations

f. sp.: forma specialis
ff. spp.: formae speciales
Fo: *Fusarium oxysporum*
Fol: *Fusarium oxysporum* f. sp. *lycopersici*
*mimp*: *miniature impala*
SIX: secreted in xylem
PDA: potato dextrose agar
ORF: open reading frame.
