# Supplementary figures and images for: Number of Candidate Effector Genes in Accessory Genomes Differentiates Pathogenic From Endophytic Fusarium oxysporum Strains
Source: Front Plant Sci. 2021 Nov 29;12:761740. doi: 10.3389/fpls.2021.761740 (PMC8666634; doi:10.3389/fpls.2021.761740)

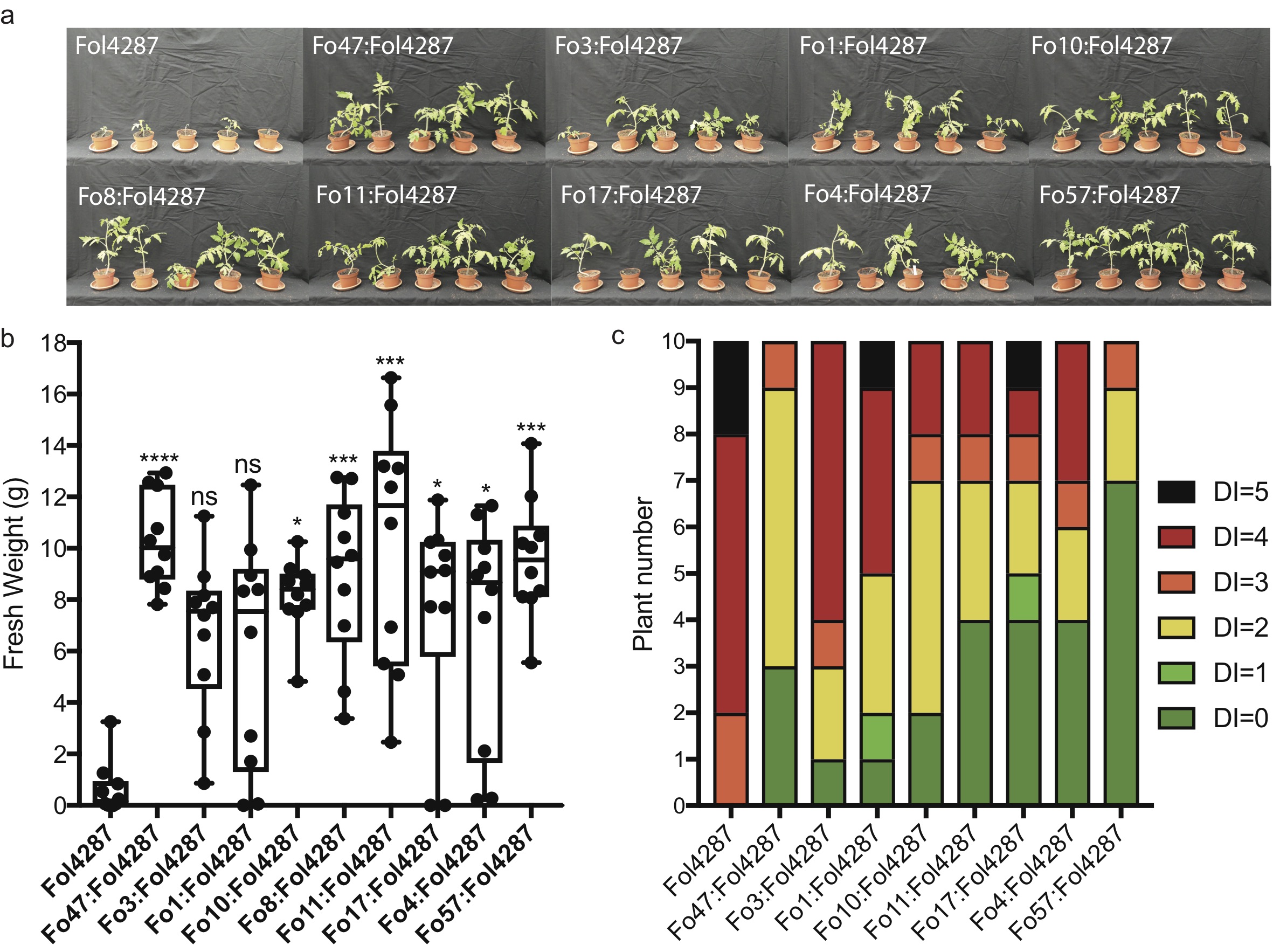

Supplement: Supplementary Figure 1 — Fo3 colonizes tomato roots poorly and does not protect against Fusarium wilt disease. (a) Pictures of representative tomato plants 3 weeks after inoculation with Fol4287 only or together with the endophytic strain Fo3, Fo1, Fo4, Fo10, Fo8, Fo11, Fo17, or Fo57. Fresh weigh above cotyledon level (b) and disease symptoms (c) were scored 3 weeks after inoculation. Fresh weight data were analyzed using Kruskal–Wallis test with a Dunn’s multiple comparisons test where Fol4287 was defined as control group. [file Image_1.JPEG]
